# THROUGH THE LENS OF THEORY: APPROACHES TO BEHAVIORAL INTERVENTION OPTIMIZATION AND EVALUATION IN AGING ADULTS

**DOI:** 10.1093/geroni/igae098.0131

**Published:** 2024-12-31

**Authors:** Ashley Curtis, Christina McCrae

**Affiliations:** Chair; Discussant

## Abstract

A shortage of geriatric mental health providers, limited efficacy of pharmacological agents for cognition, sleep and associated functioning, as well as polypharmacy-related side effects, has prompted research on behavioral interventions. Several behavioral interventions show promise for improving cognition and associated functioning, but optimization of treatments according to target end-user needs and characteristics is needed. This symposium describes psychosocial theory-based approaches for optimization and evaluation of behavioral interventions for aging adults. Dr. Ashley Curtis (Chair) will introduce the session. Paper 1 applies a biopsychosocial model to evaluation of cognitive training benefits and describes how intervention expectations and game engagement impact training benefits in middle-aged/older adults. Paper 2 discusses an iterative optimization process for a multi-modal positive psychiatry intervention for older adults that targets psychological wellness, health, and resilience. Paper 3 describes a user-centered mixed-methods evaluation of needs/preferences of grandparents raising grandchildren for behavioral sleep treatment and proposes recommendations for optimization of cognitive behavioral therapy for insomnia (CBT-I). Paper 4 describes mixed-methods optimization of web-based CBT-I for rural dementia caregivers and presents preliminary efficacy for sleep, arousal, cognition, and associated outcomes. Dr. Christina McCrae (Discussant) will integrate findings and discuss areas for future investigation. In sum, this symposium applies a theoretical lens to the optimization and evaluation of behavioral interventions for improving cognition, sleep and associated functioning in mid-to-late life. Thus, this session will provide recommendations for precision medicine-based tailoring of digital/multi-modal interventions that can be easily disseminated (online/in the community), to maximize improvement of psychological and behavioral wellbeing of aging adults.

